# Implications of the expression of *Enterococcus faecalis* citrate fermentation genes during infection

**DOI:** 10.1371/journal.pone.0205787

**Published:** 2018-10-18

**Authors:** Gabriela P. Martino, Cristian E. Perez, Christian Magni, Víctor S. Blancato

**Affiliations:** 1 Laboratorio de Fisiología y Genética de Bacterias Lácticas. Instituto de Biología Molecular y Celular de Rosario (IBR), Concejo nacional de investigaciones científicas y tecnológicas (CONICET)–UNR, Rosario, Argentina; 2 Laboratorio de Biotecnología e Inocuidad de los Alimentos. Facultad de Ciencias Bioquímicas y Farmacéuticas (FBioyF)–Municipalidad de Granadero Baigorria. Universidad Nacional de Rosario (UNR), Granadero Baigorria, Argentina; 3 Biotecnología de los Alimentos, LCTA, FCByF–UNR, Rosario, Argentina; The University of Sydney, AUSTRALIA

## Abstract

Citrate is an ubiquitous compound in nature. However, citrate fermentation is present only in a few pathogenic or nonpathogenic microorganisms. The citrate fermentation pathway includes a citrate transporter, a citrate lyase complex, an oxaloacetate decarboxylase and a regulatory system. *Enterococcus faecalis* is commonly present in the gastro-intestinal microbiota of warm-blooded animals and insect guts. These bacteria can also cause infection and disease in immunocompromised individuals. In the present study, we performed whole genome analysis in *Enterococcus* strains finding that the complete citrate pathway is present in all of the *E*. *faecalis* strains isolated from such diverse habitats as animals, hospitals, water, milk, plants, insects, cheese, etc. These results indicate the importance of this metabolic preservation for persistence and growth of *E*. *faecalis* in different niches. We also analyzed the role of citrate metabolism in the *E*. *faecalis* pathogenicity. We found that an *E*. *faecalis* citrate fermentation-deficient strain was less pathogenic for *Galleria mellonella* larvae than the wild type. Furthermore, strains with deletions in the oxaloacetate decarboxylase subunits or in the α-acetolactate synthase resulted also less virulent than the wild type strain. We also observed that citrate promoters are induced in blood, urine and also in the hemolymph of *G*. *mellonella*. In addition, we showed that citrate fermentation allows *E*. *faecalis* to grow better in blood, urine and *G*. *mellonella*. The results presented here clearly indicate that citrate fermentation plays an important role in *E*. *faecalis* opportunistic pathogenic behavior.

## Introduction

**Fig 1:**
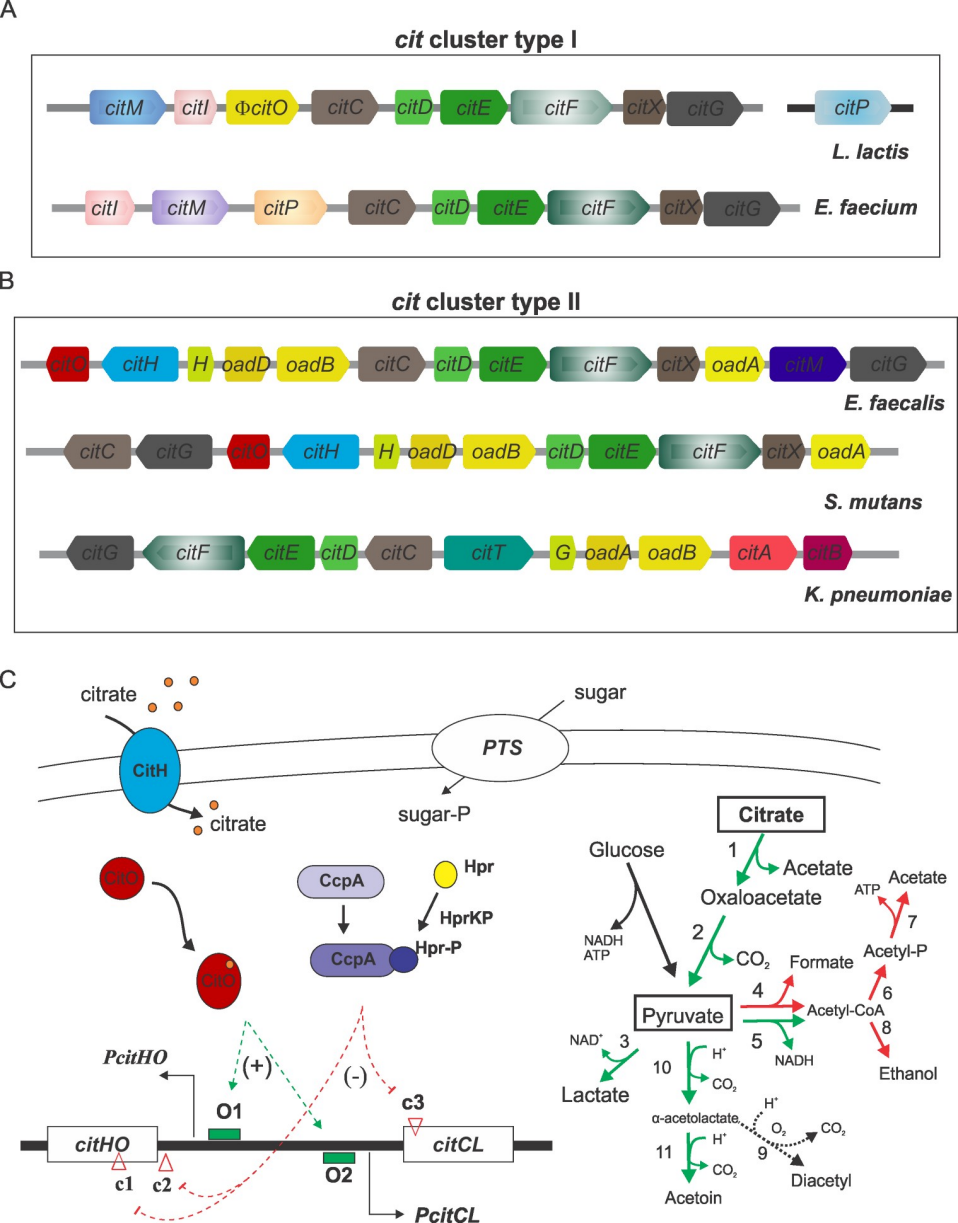
Citrate gene clusters and metabolic pathway. (A) and (B) Scheme of citrate metabolic pathway gene organization. *citM*, soluble oxaloacetate decarboxylase; *citI*, transcriptional regulator (deoR family); *citC*, *citDEF*, *citX*, and *citG*, citrate lyase subunits and accessory proteins; *citP*, citrate transporter (2-HCT family); *ɸcitO*, pseudo gen; *citO*, transcriptional regulator (gntR family); *citH*, citrate transporter (citMHS family); H (*oadH)*, *G (oadG)*, *oadD*, *oadB* and *oadA*, subunits of the membrane-bound oxaloacetate decarboxylase; *citT*, citrate transporter (2-HCT family); *citAB*, two-component signal transduction system. (C) Citrate and pyruvate pathways and their regulation in *E*. *faecalis*. Enzymes involved in citrate metabolism: 1, citrate lyase; 2, oxaloacetate decarboxylase. Enzymes involved in pyruvate metabolism: 3, lactate dehydrogenase; 4, pyruvate formate lyase; 5, pyruvate dehydrogenase; 6, phosphotransacetylase; 7, acetate kinase; 8, alcohol dehydrogenase; 9, non-enzymic oxidative decarboxylation, 10, α-acetolactate synthase and 11, α-acetolactate decarboxylase. Green and red arrows indicate induced or repressed steps (respectively) during growth in blood or urine [3, 4]. O1 and O2 binding sites of the activator CitO; c1, c2 and c3 binding sites of CcpA (cre sites).

Since all living organisms contain a certain intracellular level of citrate, this organic acid is commonly found in nature. In addition, citrate is extensively used as preservative in food and beverages. Different bacteria are able to utilize citrate via the citrate lyase pathway. This metabolism consists primarily of a transport system, which incorporates citrate into the cell, a citrate lyase complex (CL), which disrupts the molecule and an oxaloacetate decarboxylase enzyme (OAD), which produces pyruvate as a final product. Expression of the complete route is also subjected to fine regulation through different systems (Fig 1) [1, 2]. All of the proteins responsible for this pathway are encoded in gene clusters named *cit*; two types of these were identified in bacteria during the past years.

The Type I gene cluster was found in species of *Lactococcus lactis* [2, 5], *Weissella paramesenteroides* [6], *Leuconoctoc ssp*. [7] and *Enterococcus faecium* [8]. It is characterized by the presence of the *citI* activator gene (deoR family) [6], the *citM* gene encoding for a soluble oxaloacetate decarboxylase (malic enzyme family) [9, 10] and the *citP* transporter gene (member of the 2-hydroxy-carboxylate transporter family (2-HCT)) [1, 11] (Fig 1A).

The second type, Type II, is disseminated in Gram negative as well as Gram positive microorganisms. Presence of the *oad* genes, which code for a membrane bound oxaloacetate decarboxylase complex (mOAD) differentiates both clusters (Fig 1B).

The cluster present in enterobacteria is depicted for *Klebsiella pneumoniae* in Fig 1 and is similar to the one found in *Vibrio cholerae* [1]. In this microorganism, mOAD is composed of three subunits (OadA, OadB and OadG). The OadA subunit is biotinilated and soluble, while the OadB is a membrane-bound Na^+^ pump. The OadG subunit is proposed to be involved in the OAD complex assembly and stabilization. The stoichiometry of the decarboxylase complex was recently determined to be α, β, γ 4:2:2 in *V*. *cholerae* [12, 13]. In this microorganism, pyruvate molecules are then converted to less acidic compounds, such as acetoin or 2,3-butanediol [14]. In both enterobacteria, a two component system, CitAB, is involved in the regulation of this pathway expression.

In Gram positive bacteria, the Type II gene cluster has been described in several firmicutes species, such as *Streptococcus mutans*, *Lactobacillus casei* and *Enterococcus faecalis*. In *S*. *mutans*, citrate is taken up from the medium in the presence of Fe^3+^, and its degradation is directed to the production of aspartate. However, at low pH, its metabolization can inhibit growth and survival of this microorganism [15]. In *L*. *casei*, citrate is transported inside the cells by a member of the CitHMS family associated to Ca^2+^. Then, it is metabolized to form pyruvate through the mOAD activity, yielding acetate and acetoin as final products. In this case, the microorganism benefits from citrate consumption during sugar fermentation in stationary growth phase by generating a homeostatic effect in the intracellular pH [16].

In *E*. *faecalis*, a complete characterization of citrate transport [17] and fermentation was described [18]. In this bacterium, binding of the regulator CitO (a GntR superfamily member) to its target sequences located upstream of the *cit* cluster promoters activates catabolic and transporter genes in response to citrate available in the surrounding medium [18, 19]. Citrate transport in *E*. *faecalis* is carried out by CitH, a CitMHS family transporter which catalyzes proton motive force-driven uptake of the Ca^2+^–citrate complex [17]. On the other hand, expression of this metabolic route is repressed by PTS-sugars through CcpA-dependent and independent mechanisms [20] (Fig 1C). As observed in *V*. *cholerae*, the *E*. *faecalis* mOAD complex is composed of four subunits: the carboxyl transferase OadA, the Na^+^ membrane pump OadB, the biotin acceptor protein OadD and the novel subunit OadH, proposed as a functional homologous to OadG [21]. Activation of the citrate degradation pathway during cheese ripening increases the concentration of pyruvate that could be condensed to produce α-acetolactate by the α-acetolactate synthase enzyme (ALS). Then, α-acetolactate can be converted to acetoin by the activity of α-acetolactate decarboxylase, or to diacetyl in a nonenzymatic oxidative decarboxylation reaction. The volatile compounds produced (specially diacetyl), can help in the development of cheese aroma and flavor [22] (Fig 1C).

In the last decades, *E*. *faecalis* has been recognized as one of the leading causes of hospital-acquired diseases in the United States and Europe [23]. Indeed, this microorganism can infect the bloodstream, urinary tract, endocardium and biliary tract [24, 25]. Because of its innate and acquired resistance to many antibiotics, *E*. *faecalis* infections are progressively becoming more difficult to treat [23]. For this reason, new insights into the field of enterococci infection prevention are needed, and continuous knowledge generation regarding virulence-associated factors is mandatory. Well characterized virulence factors include the cytolysin CylL, the aggregation substance Agg, the metalloendopeptidase GelE, the extracellular surface protein Esp, the cell surface protein EfaA, the serine protease SprE, the adhesion to collagen protein Ace, fibrinogen Ebp and collagen Acm [26]. Experiments with animal infection models are often useful for identification of differentially expressed genes that could act as virulence traits or fitness factors [27]. Despite this, infection mechanisms, especially the transcriptional modulation occurring in living hosts, are still poorly understood.

In this study, we analyzed the role of citrate fermentation in the pathogenicity of *E*. *faecalis* using the insect *G*. *mellonella* as infection model. Citrate metabolism deficient-*E*. *faecalis* JH2-2 strains resulted less virulent than the wild type, suggesting that this process could be important for this bacterium opportunistic behavior. We also found that an active citrate metabolism allows *E*. *faecalis* to grow better in blood and urine, where citrate is present. Furthermore, an α-acetolactate synthase-deficient strain was also less virulent than the wild type suggesting that the increase of internal and external pH provided by pyruvate degradation could promote *E*. *faecalis* survival during infection.

## Materials and methods

### Bioinformatic analysis

*Enterococcus* whole genome sequences were downloaded from the NCBI Refseq database (December 2017). Duplicated genomes were detected and removed prior to analysis. Proteins belonging to *E*. *faecalis* TX4000 citrate metabolism were used as queries in local tblastn searches. Cut-off values for searches in *Enterococcus* genomes were set to 70% of sequence coverage with >70% or 57% amino acid identity for CitE and CitF, respectively. Cut-off values for searches carried out in *E*. *faecalis* genomes were 70% of sequence coverage with >85% amino acid identity.

### Bacterial strains and cultures

Bacterial strains and plasmids used in this study are listed in Table 1. *E*. *coli* strain DH5α was used as an intermediate host for cloning, and *E*. *coli* EC101 was used as host for pGhost9 constructs. *E*. *coli* strains were grown at 37° C under aerobic conditions in Luria-Bertani medium (LB), or on LB agar plates. Ampicillin (100 μg/ml), or erythromycin (150 μg/ml, 250 μg/ml) were included in the medium to select cells harboring ampicillin- or erythromycin-resistant plasmids.

**Table 1:** Plasmids and strains used in this study.

Plasmid or *S*train	Description	Source or Reference
pGhost9	Thermosensitive plasmid carrying erythromycin resistance.	[28]
pmCit	pGh9-derivative carrying a 417 bp *oadH-D* fragment.	This work
pTLGR	Promoterless vector which allows gfp and cherry transcriptional fusion construction.	[29]
pTLGR-Pcit	pTLGR carrying citrate promoters.	This work
pBM01	pUC18 derived plasmid with chloramphenicol resistance and Rep264 replicon.	[30]
pBM02	Shuttle vector carrying chloramphenicol resistance, Rep264 replicon, pUC18 replicon and PcitM promoter with *Nco*I cloning site.	[30]
pBV153	pBM01-derived plasmid with PcitM promoter and *Nde*I cloning site.	This work
pOadA	pBV153-derived plasmid for expression of *oadA*.	This work
*E*. *faecalis*		
JH2-2	Fus^r^ Rif^r^; plasmid-free wild type strain.	[31]
JH2-2/ pTLGR	JH2-2 carrying fluorescent reporter plasmid	This work
JH2-2/ pTLGR-Pcit	JH2-2 carrying fluorescent reporter plasmid with citrate cluster promoter.	This work
JH2-2-Cit^-^	*JH2-2 oadD*::*pGhost9; citrate* metabolism defective strain (Cit^-^)	This work
JH2-2-OadA^-^	*JH2-2 ΔoadA*.	[21]
JH2-2-OadA^-^/pOadA	JH2-2 *ΔOadA/ pOadA*.	This work
JH2-2-OadB^-^	*JH2-2 ΔoadB*.	[21]
*E*. *coli*		
DH5α	*F*^*−*^ *ϕ80d/lacZΔM15 Δ(lacZYA-argF) U169 recA1endA1hsdR17 (r*^*−*^_*K*_, *m*^*+*^_*K*_ *) phoA supE44 λ- thi-1 gyrA96 relA1*	
EC101	*Kan*^*r*^ *supE thi (lacproAB) (F’ traD36 proAB lacI*^*q*^ *ZΔM15) repA*	[32]

*E*. *faecalis* strains were routinely grown at 37° C without shaking in 100 ml sealed bottles filled with 20–50 ml of LB medium containing 0.5% w/v glucose (LBG). Erythromycin (5 μg/ml, 250 μg/ml), or chloramphenicol (10 μg/ml) were added when appropriate.

For urine or blood growth experiments, *E*. *faecalis* strains were grown overnight in LB supplemented with glucose 0.5% (LBG), at 37° C. Cultures were subsequently diluted 100 x in 10 ml pre-warmed LBG, and further incubated at 37° C. When an OD_600_ value of 0.1 was reached, cultures from each strain were centrifuged (10000 x g for 2 min) and resuspended in sterile human urine [4] or defibrinated mouse blood [3] and incubated at 37° C. Samples were collected at different times to determine CFU/ml or prepared for observation by fluorescence microscopy.

### Construction of *E*. *faecalis* JH2-2 mutant strains

The *E*. *faecalis* JH2-2-Cit^-^ strain was constructed by interrupting the *oadD* gene by a single recombination event using the thermosensitive vector pGhost9 [28]. A fragment of 417 bp comprising the 3´end of *oadH* and the 5´ end of *oadD* was amplified by PCR using chromosomal DNA of *E*. *faecalis* JH2-2 as template. Forward primer EfoadH (5´-GGGCTGTCAGAAGAAGCTTAGCTAGTTG-3´) introduced a *Hind*III site, while reverse primer EfoadD_Up (5´-ACATGAATTCCTGTTACCGTACCTG-3´) introduced an *Eco*RI site. After digestion, the PCR product was ligated into the corresponding sites of the pGhost9 vector. The resulting plasmid, named pmCit (Table 1), was introduced into *E*. *coli* EC101, isolated, and then electroporated into the *E*. *faecalis* JH2-2 strain. Citrate-deficient strain JH2-2-Cit^-^ (Table1) was constructed as described in [18], and insertion verified by PCR.

pBV153 plasmid for complementation was constructed as follows. The promoter region PcitM was PCR amplified with primers EcoPr (5´- GTAGATGAATTCCAAAAAAATAATGCA-3´) and PrNde (5´- GATCAACCATATGTCTTCTTTCCTAAT-3´) using pBM02 plasmid as template (Table 1) [30]. The PcitM PCR product was cloned in pGemT-easy (Promega), this resulting plasmid was subsequently digested with *EcoR*I, and the released fragment was ligated in the *EcoR*I site of pBM01 plasmid (Table 1) [30]. Desired PcitM orientation was determined by restriction analysis and sequencing in the University of Maine, DNA sequencing Facility, US DNA Sequencing.

The JH2-2-OadA^-^/pOadA was constructed by electroporation of a pOadA plasmid into the JH2-2-OadA^-^ strain [21]. Briefly, *E*. *faecalis* JH2-2 *oadA* gene was PCR-amplified with primers EfOadA-*Nde*I (5´-AGCCATATGAGTAAAAAAATTCGTTTTAC-3´) and EfOadA-*Xba*I (5´-CGGTCTAGATGCCTGTTCTATTCTG-3´). After digestion, the PCR product was ligated into *Nde*I-*Spe*I digested pBV153 plasmid (Table 1), thus allowing the constitutive OadA expression for *trans*-complementation. Correct amplification of *oadA* was confirmed by sequencing.

### Construction of a fluorescent reporter plasmid

The plasmid bearing the promoter-gfp and -cherry transcriptional fusion is derived from the pTLGR plasmid [29]. The promoter region of the citrate divergent operons *citHO* and *oadHDB-citCDEFX-oadA-citMG*, was amplified by PCR with primers BamHprom-Up (5´-AGGGGATCCATTACTAAAGATGTAAAC-3´) and BamHprom-Lo (5´-TTAGGATCCTAAATATTCTTTCCC-3´) which introduced a *BamH*I restriction site, using chromosomal DNA of *E*. *faecalis* JH2-2 as template. The fragment was digested with *BamH*I and ligated in the same site of pTLGR plasmid. Fragment orientation was determined by PCR, and confirmed by sequencing. After isolation, the pTLGR-Pcit plasmid was electroporated into the *E*. *faecalis* JH2-2 strain.

### Infection and survival experiments

*E*. *faecalis* strains were grown overnight in LB medium without shaking at 37° C. Then, the bacterial cultures were diluted in 50 ml of LB medium to a final OD of 0.1 and grown at 37° C without shaking, until exponential phase was reached (4,5h). The cultures were centrifuged, resuspended in PBS 1X + 20% glycerol and frozen at -80° C. Inoculums prepared in this way were plated to determine CFU/ml. Larvae were inoculated by direct injection into the hemocoel using a Hamilton syringe 705 equipped with a repeating dispenser. Each larvae group of 16 individuals was inoculated with a fixed CFU/larva ratio ranging from 9 x 10^6^ to 6 x 10^7^. After injection, larvae were incubated at 30° C. Survival of the individuals was monitored every two to four hours, by direct observation and gently touching non-motile larvae to evidence movement response or confirm death. Survival of the larvae group was evaluated until 72 h post-infection.

### Determination of *in vivo* bacterial loads

For bacterial count in hemolymph, 45 larvae were inoculated with a total of 9 x 10^6^ CFU/larva for each strain. Fifteen larvae were separated from the group at 0, 24 and 48 h post-inoculation, three sub-groups for each time were formed hemolymph extracted, pooled together and suspended in cold Insect Physiological Saline (IPS) buffer (150 mM sodium chloride, 5 mM potassium chloride, 10 mM Tris HCl pH 6.9, 10 mM EDTA and 30 mM sodium citrate) [33] with Triton X-100 0.03% v/v. Pools were vortexed, incubated at room temperature for 15 seconds and then plated in LBG for CFU counting. The assay was carried out in duplicate, with three technical replicates for each time point.

### Hemocyte collection and fluorescence assays

At different times, hemolymph was extracted and pooled from larvae inoculated with *E*. *faecalis* strains JH2-2/pTLGR or JH2-2/pTLGR-Pcit carrying the fluorescent plasmid. Pools were diluted 1/100 in cold IPS, centrifuged at 700 x g, washed twice and finally resuspended in PBS 1X containing glucose 5 mM, MgCl_2_ 1 mM, and CaCl_2_ 0.5 mM [33]. Samples were fixed with formaldehyde and slides mounted in VectaShield (Vector Laboratories, BIOARS S.A., Argentina). Images were acquired in a Nikon E600 microscope, using a 60×1.4 WD Plan-ApoVC objective with a Nikon DXM1200 digital camera using ACT-1 software. Fiji software [34] was used to pseudocolor images.

### Data analysis

Data analysis was done using R software. Survival curves were constructed according to the Kaplan-Meier method, using the LogRank and Holm-Sidak tests [35] for multiple comparisons.

To determine differences between bacterial count in blood, urine and hemolymph, a one-way analysis of variance was performed for each time point and the Tukey´s test was selected for multiple comparisons. In our model the fixed effect are the strains and random effect is the time. For the hemolymph experiment the experimental unit is the pool of 5 larvae; for the experiment with blood and urine the experimental unit is each tube where bacteria was grown. CFU/ml data was converted to Log(CFU/ml) and then analysis was performed, Normality of the Log(CFU/ml) data was confirmed with Shapiro-Wilks test. P value was set at 0.05 in all cases.

## Results

### Diversity of citrate fermentation pathways in the *Enterococcus* genus

Citrate fermentation-associated genes were searched *in silico* in the *Enterococcus* genomes available at the NCBI RefSeq database. A total of 1478 genomes comprising different enterococci species were selected for further analysis. Using the citrate lyase *citE and citF* genes from *E*. *faecalis* TX4000 strain, we found that more than half of the genomes (758) encoded these genes. Consequently, we focused the search on the *E*. *faecalis* species. From 501 available genomes, we found that citrate metabolism is present in almost all of them, since 500 genomes encoded the citrate lyase genes. Next, all the genes of the *cit* cluster in the *E*. *faecalis* TX4000 strain were found by BLAST searches in the 500 *E*. *faecalis cit*^+^ genomes analyzed indicating that citrate metabolism is highly conserved among *E*. *faecalis* strains.

Gene context analysis of *Enterococcus* genus representative strains revealed that Type I citrate metabolism cluster is present only in *E*. *faecium* [8, 36], *E*. *ratii* and *E*. *durans* species. On the other hand, Type II was found in at least fifteen species: *E*. *faecalis*, *E*. *faecium*, *E*. *casseliflavus*, *E*. *caccae*, *E*. *haemoperoxidus*, *E*. *moraviensis*, *E*. *silesiacus*, *E*. *phoeniculicola*, *E*. *gallinarum*, *E*. *flavescens*, *E*. *saccharolyticus*, *E*. *durans*, *E*. *pallens*, *E*. *columbae* and *E*. *malodoratus*.

These results suggest that this metabolism could be playing an important role in these microorganisms ability to grow, persist or colonize different niches. In particular, the genes and arrangement described for the *E*. *faecalis* JH2-2 strain [18] (Fig 1B) are conserved in all of the strains, despite their different origins (hospital, water, food or plant). In all of the *E*. *faecalis* analyzed genomes, the transcriptional activator CitO, responsible for the induction of the cluster in the presence of citrate, the citrate transporter CitH, the citrate lyase complex (CitD, CitE, CitF) and its accessory proteins (CitC, CitG and CitX), the four subunits of the membrane OAD (*OadH*, *OadA*, *OadD* and *OadB*) and the soluble OAD (CitM) were found encoded.

### *G*. *mellonella* as a model to study the role of citrate fermentation in virulence

The connection between citrate metabolism and aroma compound production pathways has been extensively studied as well as the regulatory mechanisms involved [8, 18–22, 37]. Although many *E*. *faecalis* strains are known opportunistic pathogens, the relationship between citrate utilization route and pathogenicity has not been previously analyzed.

**Fig 2:**
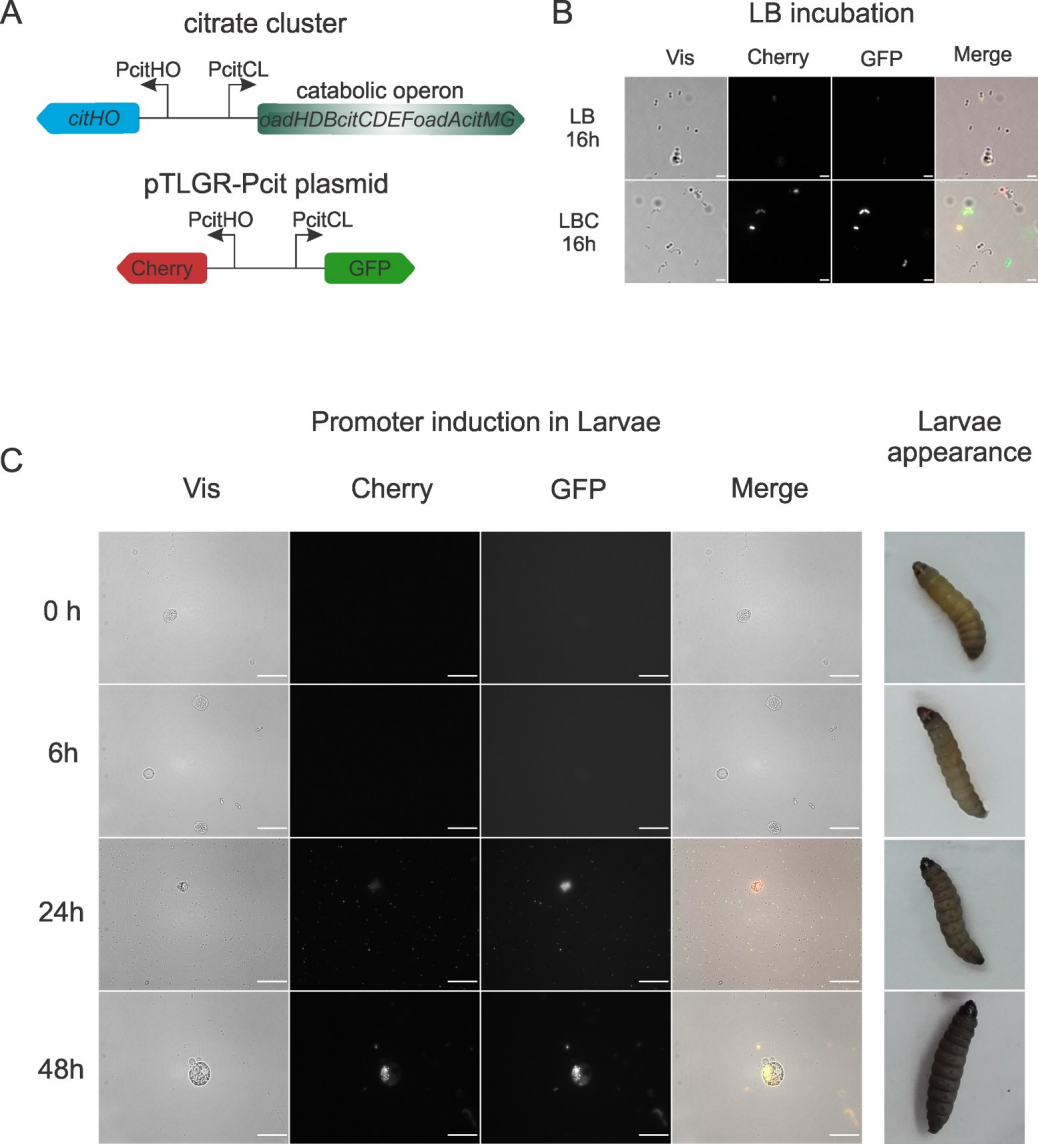
Induction of *cit* promoters in *G*. *mellonella*. (A) Scheme of citrate metabolism genes and fluorescent reporter plasmid pTLGR-Pcit used for microscopy. Fluorescence microscopy at different time points of *E*. *faecalis* JH2-2 cells harboring pTLGR-Pcit plasmid grown in LB and LB with 0.5% citrate (LBC), scale bar 5 μm (B); or *G*. *mellonella* hemolymph extracted at different time points after *E*. *faecalis* JH2-2 pTLGR-Pcit infection, scale bar 50 μm (C). Representative individuals of inoculated larvae are shown in (C). Two independent experiments were carried out and representative images acquired are shown.

Thus, *G*. *mellonella* was selected as a suitable infection model to investigate this link. Use of this moth´s larvae as an alternative model has proven useful in simple infection experiments, giving fast and reliable results, which at the same time correlate with those obtained with more traditional models [27, 38–40]. Thus, to determine if genes responsible for citrate fermentation are expressed in *E*. *faecalis-*infected larvae, a fluorescent probe was used. The divergent promoter region of the *cit* cluster was cloned in the pTLGR fluorescent reporter plasmid [29], generating the pTLGR-Pcit construct (Table 1). In pTLGR-Pcit, PcitCL (the promoter of the catabolic operon) controls the expression of GFP whereas PcitH (the promoter of the citrate transporter and regulator) controls the expression of the Cherry protein (Fig 2A).

*E*. *faecalis* JH2-2 strain was electroporated with pTLGR or pTLGR-Pcit plasmids. Next, *E*. *faecalis* strains harboring pTLGR or pTLGR-Pcit plasmids were grown in LB and LB with 0.5% citrate (LBC); to assess promoter induction samples were withdrawn after 16 hs of growth at 37°C and observed by fluorescence microscopy. As shown in Fig 2B, in LB a few cells showed a faint GFP and Cherry fluorescence indicating a low basal activity of the promoters [18]; on the contrary, as expected in LBC *E*. *faecalis* cells showed strong fluorescent signals, demonstrating induction of both promoters. No fluorescence was detected in the empty pTLGR plasmid (not shown).

Next, *G*. *mellonella* larvae groups were injected with 1 x 10^7^ CFU/larva of *E*. *faecalis* JH2-2 pTLGR-Pcit or pTLGR. Hemolymph of sample individuals was extracted at different time points after inoculation and fluorescent bacteria were detected. After 24 hours of inoculation (Fig 2C), evidence of active *cit* promoters was found, both free in the hemolymph and associated to hemocytes. A strong fluorescent signal is observed around the hemocytes, indicating that several bacteria are in contact with them, at 48 h. Also during the assay, melanization and deterioration of larvae health conditions was observed for individuals inoculated with *E*. *faecalis* JH2-2 pTLGR-Pcit or pTLGR strains (Fig 2C). Production of melanin by *G*. *mellonella* larvae is part of the insect innate immune response; melanin is synthesized after infection and it is often found around encapsulated microorganisms [41]. In our case, distinctive dark spots were observed after 24 h of infection while at 48h melanization extended to the rest of the larva as a consequence of infection progression. No fluorescence was detected in the hemolymph of larvae inoculated with *E*. *faecalis* JH2-2 harboring empty pTLGR plasmid (not shown).

### Citrate metabolism deficiency impairs *E*. *faecalis* virulence in *G*. *mellonella*

Taking into consideration the above data, contribution of the *cit* cluster to *E*. *faecalis* pathogenesis in this insect model was evaluated. Larvae group survival was monitored up to 72 h post-infection and resulting data were analyzed through Kaplan-Meier curves [35, 42].

**Fig 3:**
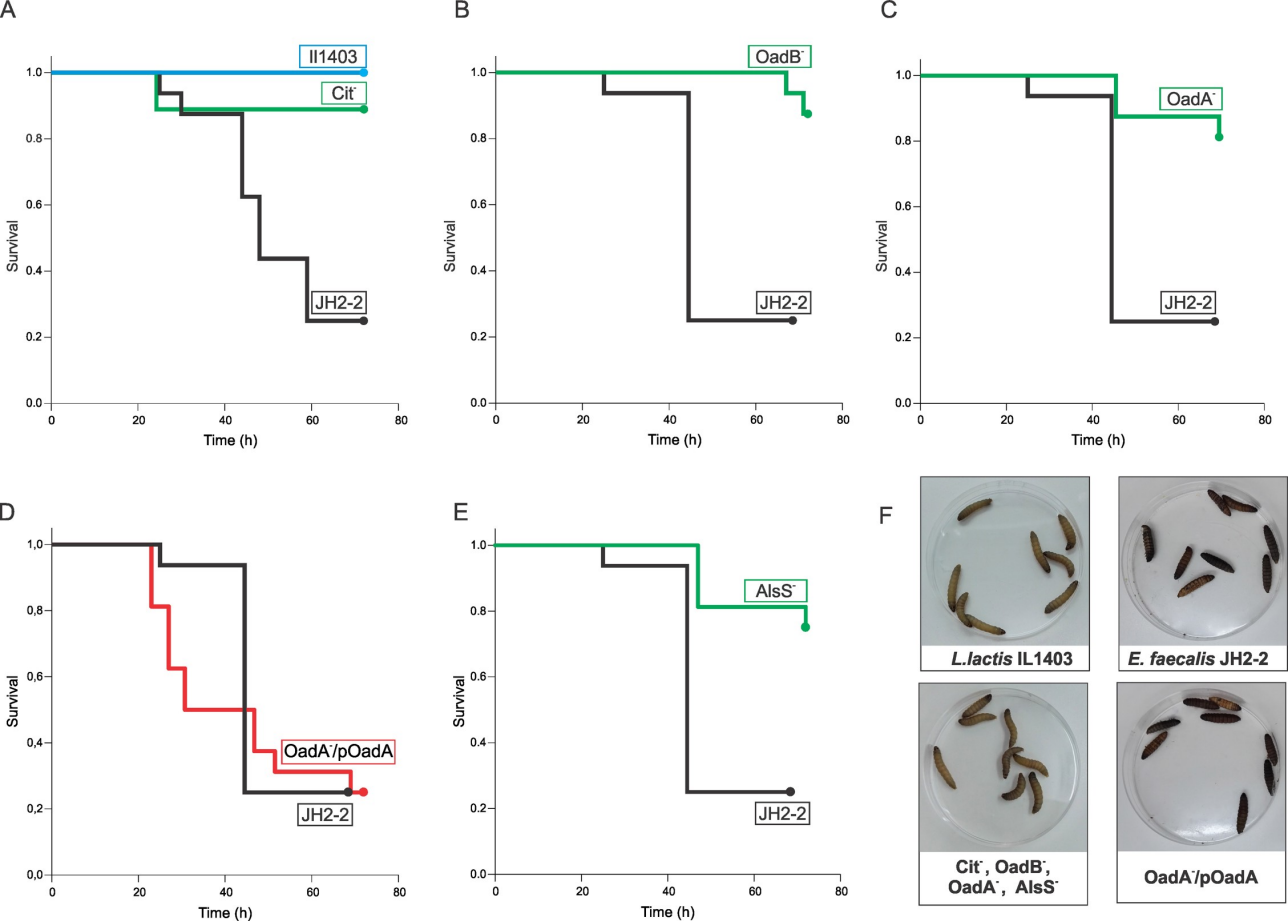
Survival of *G*. *mellonella* inoculated with different strains of *E*. *faecalis*. (A) Kaplan-Meier survival plots of *G*. *mellonella* upon injection with 4 x 10^7^ CFU/larva of *E*. *faecalis* JH2-2, or JHCit^-^. *L*. *lactis* IL1403 (4 x 10^7^ CFU/larva) was employed as a control. (B, C, D and E) Kaplan-Meier survival plots of insects upon injection with 1 x 10^7^ CFU/larva of *E*. *faecalis* JH2-2-OadB^-^, JH2-2-OadA^-^, JH2-2-OadA^-^/pOadA, or JH2-2-AlsS^-^, respectively; *E*. *faecalis* JH2-2 1 x 10^7^ CFU/larva was used as pathogenic control. (F) Images of innoculated larvae showing different degrees of disease. *G*. *mellonella* last instar larvae inoculated with *L*. *lactis* or *E*. *faecalis* citrate-deficient strains showed a typical healthy creamy color, conversely larvae infected with *E*. *faecalis* JH2-2 or *oadA*^-^ complemented strain showed different stages of disease.

*E*. *faecalis* JH2-2 injection with 4 x 10^7^ (Fig 3A) and 6 x 10^7^ CFU/larva (S1 Fig) led to high mortality rates; the first dead larva was detected after 25 and 20h (respectively) and a 30% and 5% survival rate was observed at 72h post-infection (respectively). On the other hand, *Lactococcus lactis* IL1403 (4 x 10^7^ CFU/larva) hardly appeared to be lethal (Fig 3A and 3F). No mortality was observed in PBS-injected *G*. *mellonella* larvae (data not shown). Larvae inoculated with *E*. *faecalis* JH2-2 acquired a complete melanization of the body after 48 h (Fig 3F), while *L*. *lactis* inoculated larvae remained healthy (Fig 3F).

Next, citrate fermenting-deficient *E*. *faecalis* JH2-2 strains were used to establish *cit* genes contribution to this bacterium infection capacity. A JH2-2-Cit^-^ strain, impaired at citrate use (final CFU/ml in LBC (1.47 ± 0.01) x10^8^ vs (1.89 ± 0.04) x10^8^ for the wildtype, S2 Fig), was constructed with an interruption within the *cit* gene cluster (Table 1). We carried out larvae inoculations with a total of 4 x 10^7^ CFU/larva of this strain. Despite the low survival percentage observed for the wild type (WT) strain, almost no larvae mortality was obtained over the course of a 72 h experiment for the JH2-2-Cit^-^ strain (Fig 3A). Even with a higher inoculum concentration (6 x 10^7^ CFU/larva) this mutant strain resulted barely lethal to *G*. *mellonella* (S1 Fig). A representative group of larvae inoculated with the JH2-2-Cit^-^ strain can be observed in Fig 3F. Remarkably, these low lethality levels are similar to those observed with the nonpathogenic innocuous *L*. *lactis* IL1403 strain. These findings suggest that larvae mortality induced by *E*. *faecalis* could be dependent on the presence of an active citrate metabolism.

In order to extend our knowledge about the role of citrate metabolism in *E*. *faecalis* infection of *G*. *mellonella*, mutants in the subunits of the membrane decarboxylase OAD were used. The JH2-2-OadA^-^ strain cannot metabolize citrate beyond oxaloacetate; on the contrary the JH2-2-OadB^-^ strain is capable of slowly degrading oxaloacetate to pyruvate by the action of the cytoplasmic OadAHD complex [21]. Thus, survival of *G*. *mellonella* inoculated with these strains was monitored to analyze involvement of the mOAD complex in *E*. *faecalis* virulence. Since lethality is influenced by inoculum concentration, we injected larvae with several CFU values to observe variations in strain pathogenicity. Virulence of the JH2-2-OadB^-^ strain in *G*. *mellonella* larvae was tested in a CFU range varying from 9 x 10^6^ to 3 x 10^7^ CFU/larva. This strain resulted less virulent than the WT when inoculated at CFU of 9 x 10^6^, 1 x 10^7^ and 3 x 10^7^ per larva (P<0.006), with more than 68% survival (JH2-2-OadB^-^) vs. less than 44% survival (WT) for the two lowest concentrations. Results for 1 x 10^7^ CFU/larva are shown in Fig 3B, all the CFU/larva values tested are shown in S1 Fig. The JH2-2-OadA^-^ strain also resulted less virulent than the WT in the same CFU/larva range (P<0.001), with 87.5% survival, for the two lowest concentrations. Fig 3C shows the results for the 1 x 10^7^ CFU inoculum.

To corroborate the role of citrate metabolism in *E*. *faecalis* JH2-2 virulence, the mOAD complex mutation was complemented by expressing the OadA subunit in *trans* using the pOadA plasmid (Materials and methods and Table 1). JH2-2-OadA^-^ cells harboring pOadA recovered their ability to metabolize citrate resulting in an increase in final CFU/ml counts for cells grown in LBC ((2.1 ± 0.09) x10^8^ CFU/ml vs (1.57 ± 0.02) x10^8^ CFU/ml for the mutant, S2 Fig). Once the cit^+^ phenotype was confirmed, the complemented strain (JH2-2-OadA^-^/pOadA) was used to inoculate *G*. *mellonella* larvae, again a range of CFUs were assayed. As shown in Fig 3D, JH2-2-OadA^-^/pOadA cells were as virulent as the WT (P>0.001) for the lowest concentrations (9 x 10^6^ and 1 x 10^7^ CFU/larva), with values of 3 x 10^7^ CFU/larva the complemented strain was slightly more virulent than the WT (P<0.001) (S1 Fig). A group of larvae inoculated with 1 x 10^7^ CFU/larva of the complemented strain is shown in Fig 3F where body melanization to the same extent to that of the WT strain can be observed.

Repizo *et al* [21] showed that the JH2-2-OadA^-^ strain cannot metabolize citrate and, consequently, does not show growth improvement in the presence of this compound. On the other hand, in LB growth medium supplemented with citrate, JH2-2-OadB^-^ is able to reach final OD_600_ levels similar to those of the parental strain, with a delay in the beginning of the second growth phase, *i*.*e*., when citrate pathway is induced [18]. Thus, despite the ability of JH2-2-OadB^-^ to grow in citrate-containing media (LBC), it seems that under infection conditions, the delay observed in its growth is strongly detrimental for the cells and they cannot cope with the immune system of *G*. *mellonella* as well as the WT. This leads to higher larvae survival rates. Nonetheless, citrate metabolism deficiency makes strains less virulent than the WT, confirming the observation made for the JH2-2-Cit^-^ strain.

Given the connection between aroma compound production from pyruvate and citrate metabolism (Fig 1C), an α-acetolactate synthase deficient strain (JH2-2-AlsS^-^) was also used in survival experiments and compared with the other strains tested. The JH2-2-AlsS^-^ [37] strain is unable to further convert pyruvate into α-acetolactate. As a consequence, this strain exhibits growth deficiency in the presence of pyruvate at pH 5.5, and is unable to grow at pH 4.5. This indicated that the pathway involved in aroma compound generation is an important mechanism which allows growth in acidic media [37]. Inoculums of JH2-2-AlsS^-^ were prepared and used to inject *G*. *mellonella* larvae. After examination of larvae health status during 72h, KM curves were plotted (Fig 3E). When 1 x 10^7^ CFU/larva were injected, JH2-2-AlsS^-^ was less virulent than the WT (P<0.001). With a 3 x 10^7^ inoculum, the AlsS deficient strain was as virulent as the WT (P = 0.37). suggesting that the “pyruvate to acetoin” pathway could be affecting *E*. *faecalis* virulence.

### Citrate metabolism enhances *E*. *faecalis* growth in insect hemolymph, blood and urine

**Fig 4:**
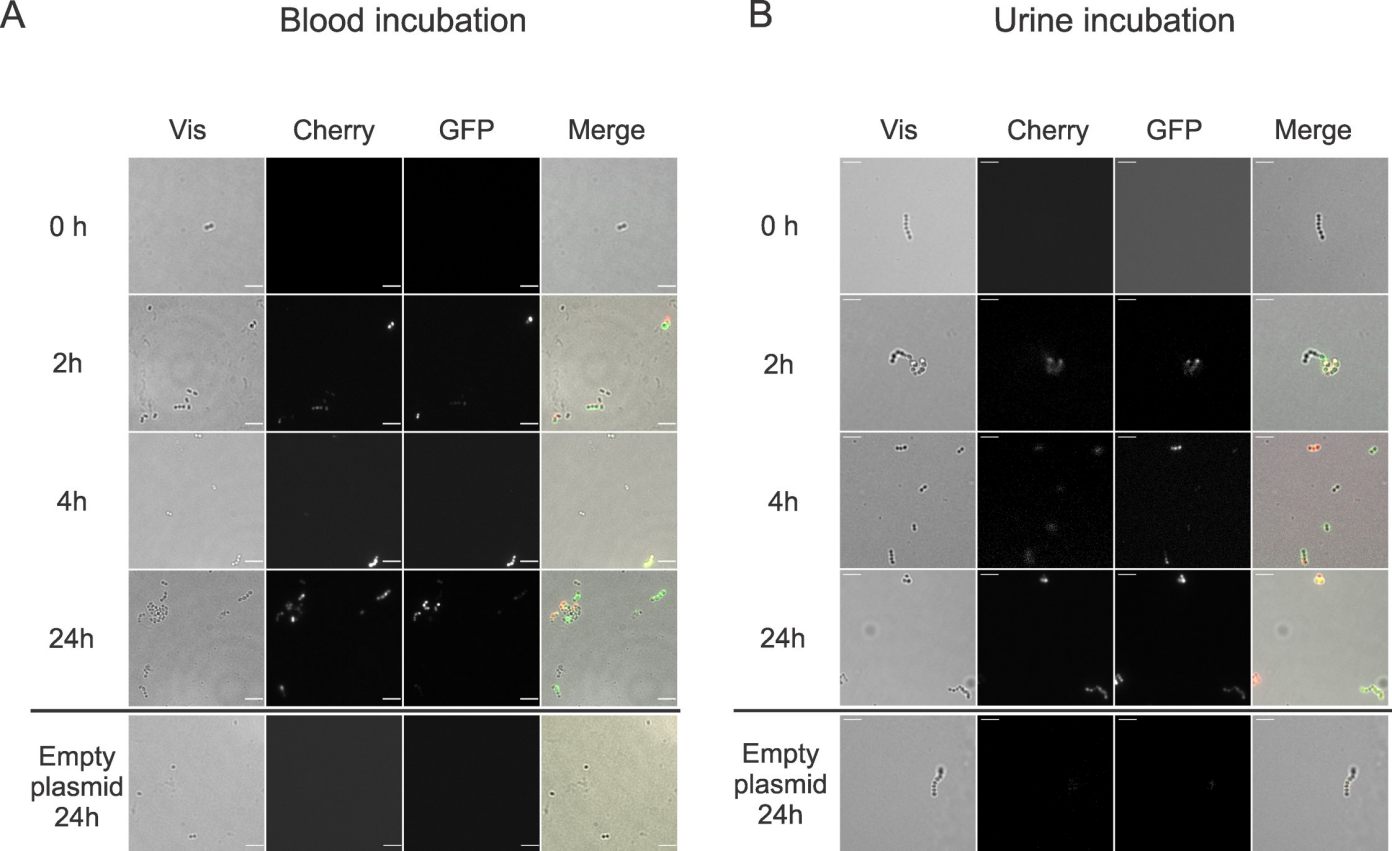
Induction of *cit* promoters in blood and urine. Fluorescence microscopy at different time points of *E*. *faecalis* JH2-2 cells harboring pTLGR or pTLGR-Pcit plasmids grown in defibrinated blood (A) or urine (B). Two independent experiments were carried out and representative images acquired of three technical replicates are shown. Scale bar 5 μm.

In previous sections we showed that citrate metabolism plays an important role during *E*. *faecalis* infection of *G*. *mellonella*. Consequently, an analysis of *cit* cluster induction in other animal fluids associated to common diseases caused by *E*. *faecalis* was performed. To this end, *E*. *faecalis* JH2-2-pTLGR or pTLGR-Pcit was grown in mouse defibrinated blood, and activity of Pcit promoters was analyzed at different time points. GFP and Cherry fluorescent signals were detected as early as 2 hours after exposition to blood (Fig 4A), indicating that both promoters were quickly induced. No fluorescence was detected at time 0 for pTLGR-Pcit or with the empty pTLGR plasmid during a 24 h assay. When *E*. *faecalis* JH2-2 pTLGR-Pcit was incubated in human urine, induction of Pcit promoters was observed after 2 h with increasing signal during the time assayed (Fig 4B). These results suggest that citrate metabolism in *E*. *faecalis* is probably being induced during blood and urine infection and could have a role in infection of higher animals as well as in *G*. *mellonella*. To confirm this hypothesis in *G*. *mellonella*, bacterial load after infection was determined. Larvae groups were infected with the WT and citrate metabolism-mutant strains. At different time points, hemolymph was extracted from sample individuals, diluted and plated onto an appropriate medium to determine total bacterial CFU/ml of hemolymph.

**Fig 5:**
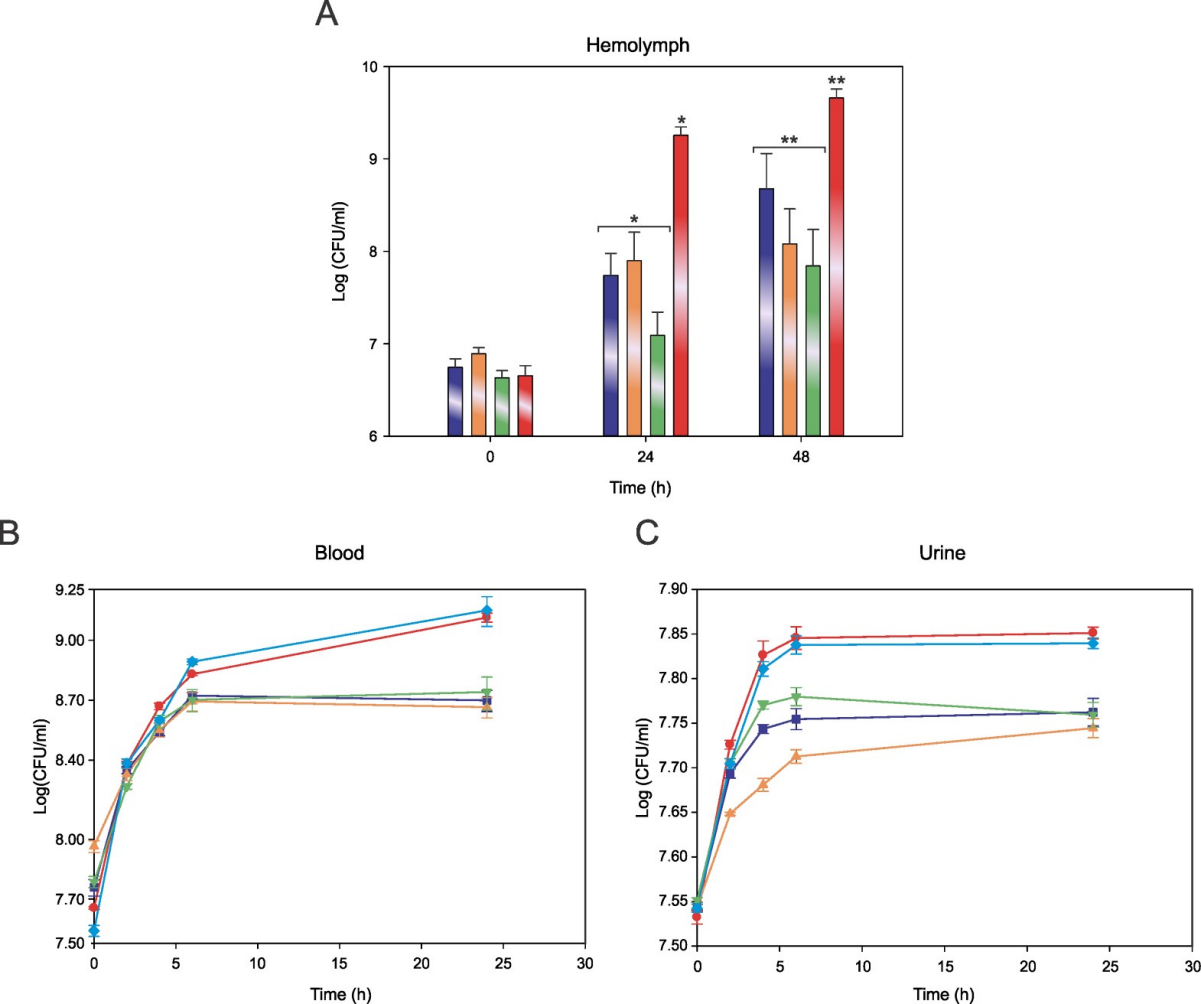
***E*. *faecalis* strains growth in *G*. *mellonela* larvae (A), blood (B) and urine (C).** (A) *G*. *mellonella* was inoculated with 9 x 10^6^ CFU/larvae. Bacterial burden was quantified using pools of hemolymph extracted from different larvae at the time points indicated. Growth was monitored by measuring colony forming units per milliliter (CFU/ml). JH2-2-OadA^-^ (purple), JH2-2-oadB^-^ (orange), JH2-2-Cit^-^ (green) and JH2-2 (red). Data points correspond to the mean ± standard error of six replicates, significative difference is indicated by * or **. (B and C) Growth of *E*. *faecalis* strains in blood and urine, respectively; *E*. *faecalis* JH2-2 (red circle), JH2-2-OadA^-^ (purple square), JH2-2-oadB^-^ (orange up triangle), JH2-2-Cit^-^ (green down triangle) and JH2-2-OadA^-^/pOadA (cyan diamond). Data points correspond to the mean ± standard error of four and three replicates, respectively.

Comparative analysis of CFU/ml at times 0, 24 and 48 h post-inoculation, allowed us to confirm that, after 24h of inoculation, the JH2-2 WT strain can effectively reach higher CFU values *in vivo* than JH2-2-Cit^-^, JH2-2-OadA^-^ and JH2-2-OadB^-^ (Fig 5A, P<0.01 for all the WT vs mutant comparisons). Moreover, at 24 and 48 h no statistically significant differences were found for the total bacterial count in the hemolymph between mutant strains (Fig 5A, P>0.4 for all the comparisons).

These results suggest that citrate metabolism-deficient strains are unable to proliferate as well as the WT in the hemolymph and are probably eliminated by the larvae immune system, thus provoking a less aggressive infection.

Since we showed that citrate genes are induced in blood, we analyzed the growth behavior of the various mutants and the OadA^-^ complemented strain to determine if their differential capacity for citrate utilization can be correlated with a differential growth. As shown in Fig 5B, no differences in growth were found among strains up to 6 h (P = 1). But, after 24 h of growth, *E*. *faecalis* JH2-2 and JH2-2-OadA^-^/pOadA reached a higher cell density than citrate metabolism mutants (Fig 5B). Statistical analysis indicated that these differences were significant (P<0.05), showing that induction of citrate metabolism could give *E*. *faecalis* WT strain an advantage in the overall growth process.

When strains were grown in urine (Fig 5C) a clear difference in growth among WT, JH2-2-OadA^-^/pOadA and mutant strains was observed at 6 h of incubation. After this point, the JH2-2-OadA^-^ and JH2-2-Cit^-^ mutants did not grow any further while JH2-2-OadB^-^, JH2-2 WT and JH2-2-OadA^-^/pOadA continued with a slow growth. Statistical analysis indicated that the differences observed were significant (P<0.05), meaning that mutant strains grow less effectively in urine than the WT or JH2-2-OadA^-^/pOadA.

## Discussion

*E*. *faecalis* is a natural member of the gastro-intestinal microbiota of warm-blooded animals and insect guts. The intrinsic ability of this bacterium to resist harsh conditions allows it to persist in hospital environments and to survive host defenses [43]. Nevertheless, many of these microorganisms are associated with food production and some strains also possess probiotic properties [22, 44, 45]. Accordingly, *E*. *faecalis* is often found in diverse types of fermented food products, making them part of the human diet around the world [22, 45]. In this context, citrate fermentation is a desired trait of lactic acid bacteria since it contributes to aroma development [2, 8, 37].

In this study, citrate fermentation operons were detected in 758 out of 1478 genomes analyzed comprising 17 species of the *Enterococcus* genus. Although citrate-negative strains were found in some of the species remarkably all of the *E*. *faecalis* genomes analyzed encoded the thirteen genes necessary for citrate fermentation. Furthermore, this Type II *cit* cluster was found conserved independently of strain origin, suggesting the importance of pathway preservation in this species.

In this work, we further demonstrate that the presence of *cit* genes contributes to the pathogenic behavior of *E*. *faecalis* in the *G*. *mellonella* model. Citrate metabolism-defective strains showed a reduced capacity of infection. When the *cit* cluster is interrupted (JH2-2-Cit^-^ strain), *E*. *faecalis* ability to metabolize citrate is abolished and, as a consequence, a remarkably lower mortality of *G*. *mellonella* was observed (Fig 3A). On the other hand, in mOAD mutants, citrate metabolism is affected to different extents; the *oadA* mutant allows the conversion of citrate to oxaloacetate but not to pyruvate (Fig 1C) [21], whereas the *oadB* mutant conserves a soluble OAD complex (OadADH) which could allow the complete conversion of citrate to pyruvate, but at a lower rate [21]. These metabolic differences between the JH2-2-Cit^-^ strain and the mOAD mutants can probably account for the observed differences in the *G*. *mellonella* survival rates after inoculation (Fig 3). In addition, an α-acetolactate synthase deficient strain (JH2-2-AlsS^-^) was also found less virulent than the *E*. *faecalis* WT strain.

Examples of metabolic pathways associated with virulence in *E*. *faecalis* are not common, Maadani *et al*. reported that an *E*. *faecalis* strain unable to metabolize ethanolamine was less virulent in the *C*. *elegans* model [46]. Ethanolamine is found in the intestine and can be used as a source of both carbon and nitrogen. The capacity to use this organic compound has been related with intestinal pathogens, for example, *Salmonella* species and *Listeria monocytogenes* [47].

Its ability to survive in hospital environments and to infect immunocompromised patients has made *E*. *faecalis* a commonly found cause of bacteremia and urinary tract infections [23–25, 48]. In fact, many of the sequenced enterococci strains available at the NCBI GenBank database were isolated from blood or urine of hospitalized patients. In this work, activation of the citrate degradative pathway was observed in larvae but also in urine and blood (Fig 4). In this media, concentration of preferred carbon sources such as glucose or fructose may fluctuate between 1 to 6 mM or 0.01 to 0.5 mM, respectively [49]. Under these conditions, only when glucose concentration reaches the higher value its repressive effect could be relevant on the *cit* cluster reducing approximately 50% the activity of PcitHO promoter and 15% the activity of the catabolic operon PcitCL promoter [20]. Nevertheless, this concentration could allow cometabolism of citrate and glucose. In urine, glucose concentration can reach 1.1 mM [49] a value too low to cause citrate pathway repression. As a consequence, citrate present in both media could be used as a carbon and energy source during *E*. *faecalis* infection.

It has been proposed that the ability of *E*. *faecalis* to cause infection would not only implicate an organized regulation of several virulence factors and expression of genetic determinants, but also an adaptation of the bacterial cell physiology during the infection process [3]. This suggests that various metabolic pathways could contribute differently to virulence and its ability to persist in diverse environments.

Transcriptomic data of *E*. *faecalis* grown in blood and urine has shown that several known virulence factors, such as the *fsrB* (EF1821), *gelE* (EF1818), *cpsC* (EF2493), *ace* (EF1099) and *efaA* (EF2076) were modulated in their expression under both growth conditions [3, 4]. Also, the data reflected the cells necessity for fast adjustments to withstand nutritional changes [3, 4]. In fact, the iron, manganese, sugar and oligo-peptide ABC-transport systems were found up-regulated. Furthermore, in agreement with our results the gene cluster responsible for citrate metabolism (EF3315-27) was up-regulated (Fig 1C). These results correlate with those obtained in a recent study of *E*. *faecalis* infection in subdermal chambers, where citrate metabolism induction was also observed [50]. Transcription of pyruvate metabolism enzymes was also modified during growth in blood and urine: α-acetolactate synthase (*alsS*, EF1213), α-acetolactate decarboxylase (*alsD*, EF1214), pyruvate dehydrogenase complex (*pdH*, EF1353-56) and lactate dehydrogenase *(ldH*, EF0255) were induced (Fig 1C); whereas phosphotransacetylase (EF0949), acetate kinase (*acK*, EF1983, pyruvate formate lyase (*pflB*, EF1613), alcohol dehydrogenase (*adhE*, EF0900) were repressed (Fig 1C). The up-regulation of citrate and some enzymes of pyruvate metabolism favor the proton consuming reactions and also contribute to increase the Acetyl-CoA pool that can be used as a precursor of the FASII pathway also up-regulated in blood and urine [3, 4].

These analyses demonstrated that *E*. *faecalis* is capable to adapt its physiology depending on its surroundings and that the induction of virulence factors is probably a piece of a larger physiological adaptation displayed when *E*. *faecalis* invades a niche as a pathogen.

In this work, we demonstrated that citrate metabolism gives *E*. *faecalis* an advantage to grow in blood and urine. We have also proved that an active citrate metabolism allows the bacterium to proliferate inside living *G*. *mellonella* larvae to higher CFU/ml hemolymph counts. This suggests that citrate metabolism is a key feature in *E*. *faecalis*, possibly allowing this microorganism to overcome adverse conditions resulting in better growth conditions.

## Supporting information

S1 Fig *G*. *mellonella* Kaplan-Meier survival plots after injection with different CFU/larvae ratios of several *E*. *faecalis* strains. Complete set of KM survival plots, using 9 x 10^6^, 1 x 10^7^, 3 x 10^7^, 4 x 10^7^ or 6 x 10^7^ CFU/larva of *E*. *faecalis* JH2-2, JH2-2-Cit^-^, JH2-2-OadA^-^, JH2-2-oadB^-^, JH2-2-OadA^-^/pOadA and JH2-2-AlsS^-^.
(TIF)

S2 Fig *E*. *faecalis* strains growth in LB medium. (A) Growth of *E*. *faecalis* JH2-2 (red circle) and JH2-2-Cit^-^ (green down triangle) strains in LB (empty symbols) or LB supplemented with citrate (closed symbols). (B) Growth of *E*. *faecalis* JH2-2-OadA^-^ (yellow up triangle) and JH2-2-OadA^-^/pOadA (cyan diamond) strains in LB (empty symbols) or LB supplemented with citrate (closed symbols). The data points correspond to the mean ± standard error of three replicates.
(TIF)

S1 Table Fig 5 raw data. (XLSX)
