# Supplementary figures and images for: Implications of the expression of Enterococcus faecalis citrate fermentation genes during infection
Source: PLoS One. 2018 Oct 18;13(10):e0205787. doi: 10.1371/journal.pone.0205787 (PMC6193673; doi:10.1371/journal.pone.0205787)

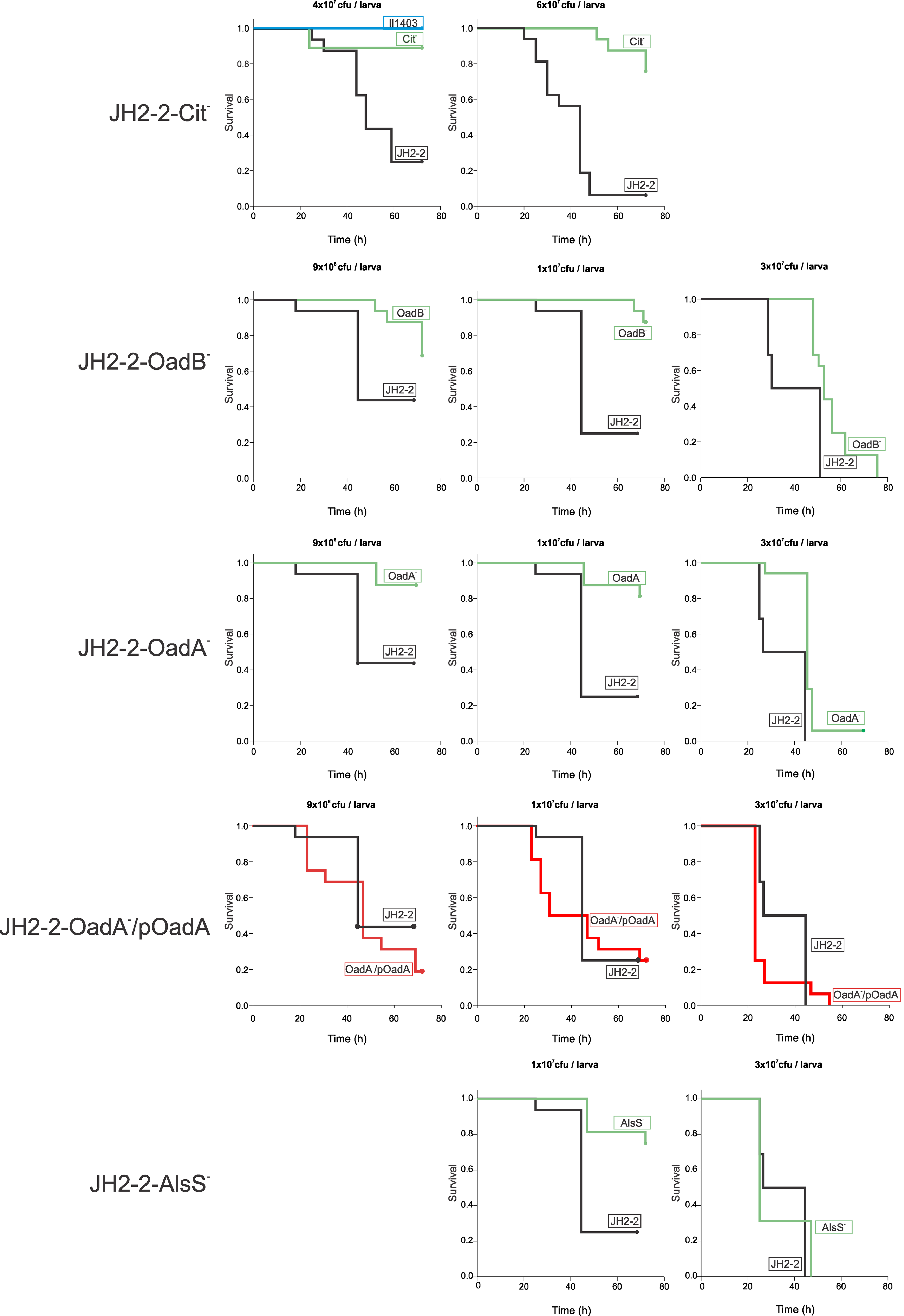

Supplement: S1 Fig — Complete set of KM survival plots, using 9 x 10^6^, 1 x 10^7^, 3 x 10^7^, 4 x 10^7^ or 6 x 10^7^ CFU/larva of E. faecalis JH2-2, JH2-2-Cit-, JH2-2-OadA-, JH2-2-oadB-, JH2-2-OadA-/pOadA and JH2-2-AlsS-. (TIF) [file pone.0205787.s001.tif]

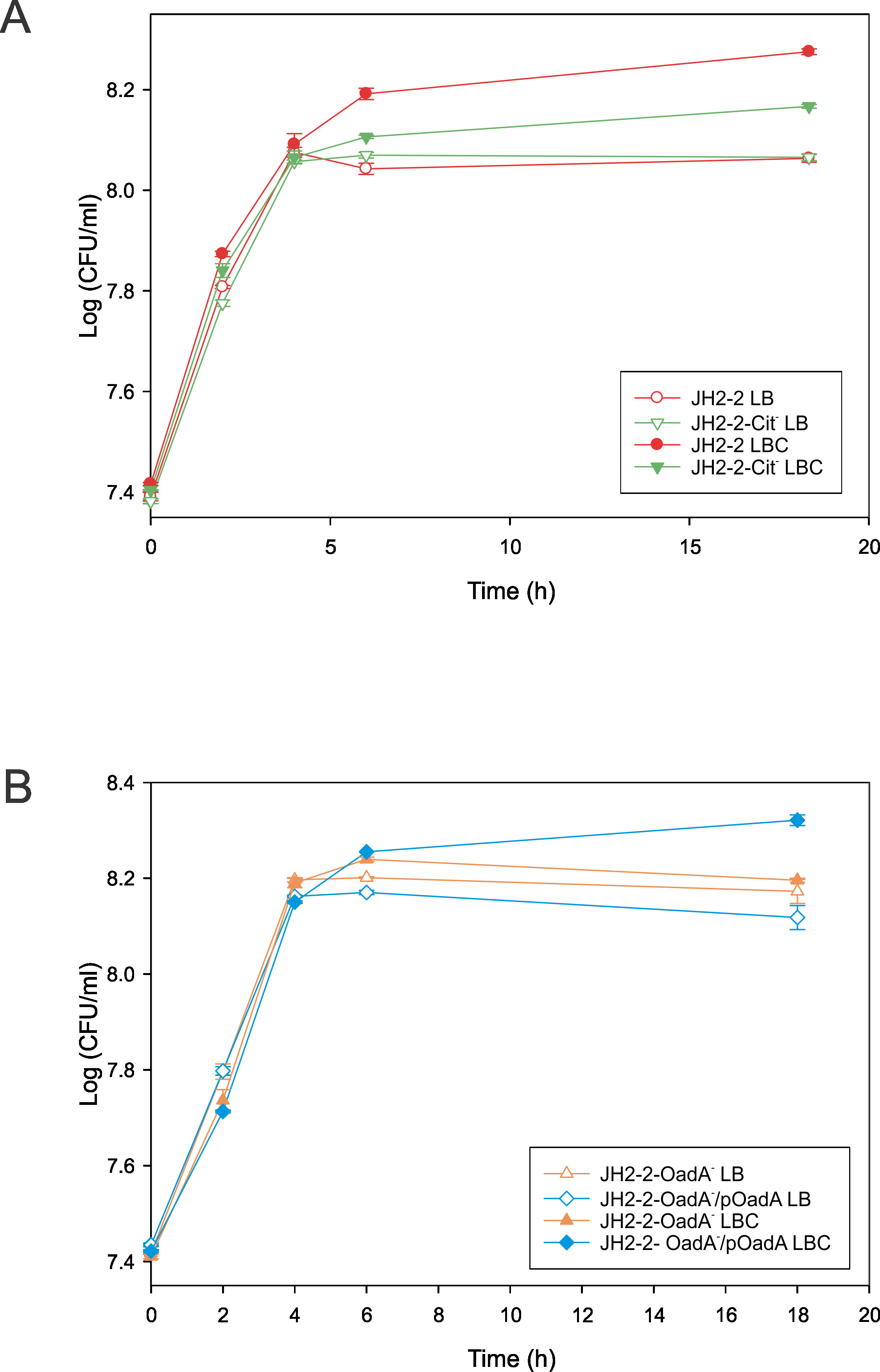

Supplement: S2 Fig — (A) Growth of E. faecalis JH2-2 (red circle) and JH2-2-Cit- (green down triangle) strains in LB (empty symbols) or LB supplemented with citrate (closed symbols). (B) Growth of E. faecalis JH2-2-OadA- (yellow up triangle) and JH2-2-OadA-/pOadA (cyan diamond) strains in LB (empty symbols) or LB supplemented with citrate (closed symbols). The data points correspond to the mean ± standard error of three replicates. (TIF) [file pone.0205787.s002.tif]
